# Using kICS to Reveal Changed Membrane Diffusion of AQP-9 Treated with Drugs

**DOI:** 10.3390/membranes11080568

**Published:** 2021-07-28

**Authors:** Jakob L. Kure, Thommie Karlsson, Camilla B. Andersen, B. Christoffer Lagerholm, Vesa Loitto, Karl-Eric Magnusson, Eva C. Arnspang

**Affiliations:** 1 SDU Biotechnology, Department of Green Technology, University of Southern Denmark, Campusvej 55, 5230 Odense M, Denmark; jlk@igt.sdu.dk (J.L.K.); caba@igt.sdu.dk (C.B.A.); 2 Division of Medical Microbiology, Department of Clinical and Experimental Medicine, Faculty of Health Sciences, Linköping University, SE-58185 Linköping, Sweden; thommie.karlsson@micromedic.se; 3 MEMPHYS, Department of Physics, Chemistry and Pharmacy, University of Southern Denmark, Campusvej 55, 5230 Odense M, Denmark; christoffer.lagerholm@imm.ox.ac.uk; 4 Wolfson Imaging Centre Oxford, MRC Weatherall Institute of Molecular Medicine, University of Oxford, Headley Way, Oxford OX3 9DS, UK; 5 Division of Inflammation and Infection, Department of Biomedical and Clinical Sciences, Faculty of Health Sciences, Linköping University, SE-58185 Linköping, Sweden; vesa.loitto@liu.se (V.L.); karl-eric.magnusson@liu.se (K.-E.M.)

**Keywords:** image correlation spectroscopy, kICS, nano-domains, diffusion, plasma membrane

## Abstract

The formation of nanodomains in the plasma membrane are thought to be part of membrane proteins regulation and signaling. Plasma membrane proteins are often investigated by analyzing the lateral mobility. k-space ICS (kICS) is a powerful image correlation spectroscopy (ICS) technique and a valuable supplement to fluorescence correlation spectroscopy (FCS). Here, we study the diffusion of aquaporin-9 (AQP9) in the plasma membrane, and the effect of different membrane and cytoskeleton affecting drugs, and therefore nanodomain perturbing, using kICS. We measured the diffusion coefficient of AQP9 after addition of these drugs using live cell Total Internal Reflection Fluorescence imaging on HEK-293 cells. The actin polymerization inhibitors Cytochalasin D and Latrunculin A do not affect the diffusion coefficient of AQP9. Methyl-β-Cyclodextrin decreases GFP-AQP9 diffusion coefficient in the plasma membrane. Human epidermal growth factor led to an increase in the diffusion coefficient of AQP9. These findings led to the conclusion that kICS can be used to measure diffusion AQP9, and suggests that the AQP9 is not part of nanodomains.

## 1. Introduction

The lipid raft hypothesis [1] is highly focused on the lipids in the plasma membrane, and its proposal initiated significant research into the lipid composition and dynamics of the plasma membrane. It is believed that lipid nano-domains are small areas in the plasma membrane (10–200 nm in diameter), enriched with cholesterol and sphingolipids [2]. Another branch of nano-domains have been defined by the actin fence model [3,4]. The fence model states that plasma membrane molecules will be caught in the spacing between the actin cytoskeleton under the plasma membrane. The discovery of nano-domains accelerated the focus on the plasma membrane, as it was now considered a more complex heterogeneous and controlled system. This resulted in hypothesizing that the plasma membrane plays a significantly more important role in the cell metabolism, and that the domains are allowing more complex processes in cell signaling [5] and trafficking [6].

In this study the membrane protein aquaporin-9 (AQP9) is studied in relation to domain formation using drugs that will perturb nanodomains. AQP9 forms a channel in the cell membrane and has an active role in cell volume regulation and migration [7,8]. Other proteins of the Aquaporin (AQP) family (AQP2, AQP3, and AQP4) has been found to form nanodomains in the plasma membrane [9,10,11,12]. However, it is not known whether AQP9 forms nano-domains in the plasma membrane.

It is very challenging to investigate nano-domains with microscopy, as they are smaller than the diffraction limit. Therefore, a common approach has been to analyze diffusion behavior with fluorescence microscopy techniques. The different types of nano-domains will all result in a decreasing diffusion coefficient for the membrane protein residing the nanodomain. The lipid-derived nano-domains will be more compact and structured, resulting in slower diffusion in these areas. A similar behavior is seen from the actin-derived domains, in which the diffusing particles will be held back by the actin skeleton for periods of time, reducing the diffusion coefficient. In order to identify nano-domains, many approaches have been used to analyze the diffusion behavior in live cells [13]. The most common method is fluorescence correlation spectroscopy (FCS). A similar group of models called image correlation spectroscopy (ICS) was initially developed to be the image counterpart to FCS. Subsequently, many new ICS methods have been developed, including the k-space ICS (kICS), which is not sensitive to blinking or photobleaching [14]. kICS is a reciprocal space derivative of the ICS method, which is carried out by Fourier-transforming the acquired images into spatial frequencies. After the transformation, a time correlation is performed, resulting in a kICS correlation function r(k,τ) seen in Equation (1), where ĩ is the intensities from the Fourier transformation and * represents the complex conjugate [15]; k represents the spatial frequencies, t represents time, and τ represents the time lag.

(1) $$r\left(k,\tau \right)=\langle ĩ\left(k,t\right)\cdot ĩ*{(k,t+\tau )\rangle }_{t}$$

The kICS correlation function is then applied to the images and analysed. With kICS it is possible to define flow velocity and diffusion coefficient both in high- and low-density samples. It has been validated that kICS can provide reliable diffusion coefficients when compared to alternative methods such as, e.g., single-particle tracking [16].

The aim of the study is to show that kICS analysis can be used to determine how nano-domain pertubing drugs affect the diffusion coefficient of AQP9 in live HEK-293 cells and determine if AQP9 resides in nanodomains.

## 2. Materials and Methods

The experiments were carried out in HEK-293 cells grown in MEM with 10% FBS and 1% Pen/Strep at 37 °C, with an atmosphere of 5% CO_2_, and stably transfected with the GFP-Aquaporin-9 (GFP-AQP9) construct by using the lentiviral expression system Lenti-X^TM^ (Clontech Laboratory Inc, Mountain View, CA, USA). The cells were treated in five different ways before imaging; (1) Untreated (UNT), (2) 3 mM Methyl-β-Cyclodextrin (MβCD) at 37 °C for 10 min, (3) 250 nM human Epidermal Growth Factor (hEGF) at 37 °C for 10 min, (4) 5 µM Cytochalasin D (CytD) added at room temperature just before imaging, and (5) 500 nM Latrunculin A (LatA) added at room temperature just before imaging. The samples were excited by a LRS-0473 473nm laser (Laserglow technologies, Toronto, ON, Canada). The images were acquired as stacks of 600 images with an Andor iXon Ultra 897 on an Olympus IX81 TIRF microscope (Olympus Life Science, Waltham, MA, USA) with a 150× and 1.45 NA objective resulting in a pixel size of about 106.7 nm, an exposure time of 30 ms and 30.5 ms between frames. The kICS analysis were performed at 5 time points at different smaller ROIs, avoiding larger intracellular background signal with a max k square at 35 similar to previous studies [12,14]. The kICS analysis and data presentation have been performed in MatLAB, while the statistical calculations have been performed using R. The statistical analysis used is a two-tailed Welch *t*-test with a confidence interval of 90%, which is acceptable in populations with low sample size.

## 3. Results and Discussion

**Figure 1:**
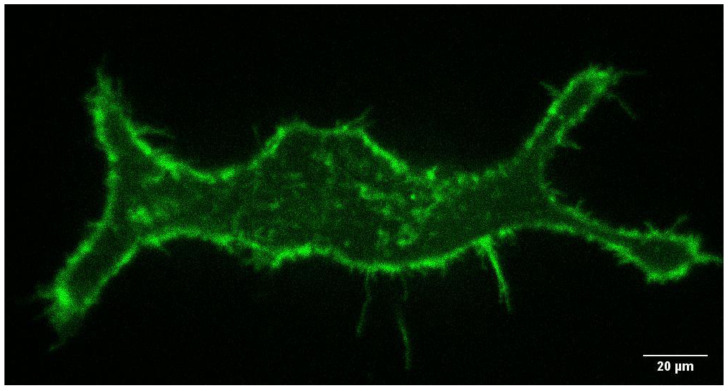
Representative TIRF-image of an untreated HEK-293 cell expessing GFP-AQP9 protein.

Live cell imaging of HEK-293 stably expressing GFP-AQP9 was conducted with and without drug treatment, and analyzed using the kICS. A representative image of a untreated cell can be seen in Figure 1. Visually, no differences on cell morphology were observed between treatments of the cells. This correlates with other studies using similar treatment conditions without changes to cell viability [17,18,19].

**Figure 2:**
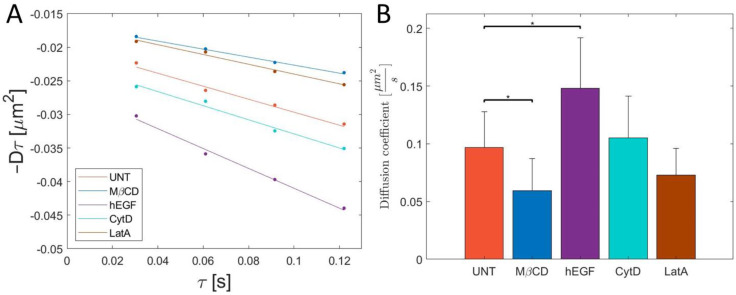
(**A**) Average linear fits from the kICS analysis depending on treatment, where the absolute value of the slope is defined as the diffusion coefficient. Dots represent the mean of all replicates at the specific timepoints. (**B**) Bar plot showing the diffusion coefficient of GFP-tagged AQP9 in live HEK-293 cells imaged by TIRF imaging and analyzed using kICS. Whiskers represent the standard deviation. * represent a statistical significance difference with a confidence interval of 90% using a two-tailed Welch *t*-test. The analysis shows that MβCD (N = 5) significantly reduces the diffusion coefficient compared to untreated (UNT) (N = 6); CytD (N = 6) and LatA (N = 4) do not have a significant effect on the diffusion coefficient; and hEGF (N = 5) significantly increases the diffusion coefficient.

The result from kICS analysis can be found in Figure 2, where Figure 2A shows the averaged linear fit from the kICS analysis and the diffusion coefficient (absolute value of the slope) is shown as a bar plot in Figure 2B.

From this, it is found that the diffusion coefficient of UNT AQP9 is 0.097 ± 0.031 µm^2^/s. This is comparable to similar studies of other aquaporins [20] at similar data sampling frequencies.

To further study the effect of lipid membrane nano-domains on AQP9 diffusion, MβCD was used to make acute cholesterol depletion in the plasma membrane. This was again analyzed with kICS, and we observed that the diffusion coefficient significantly decreased to 0.06 ± 0.028 µm^2^/s subsequent to treatment with MβCD. This may suggest AQP9 is not present in nano-domains, as an increase in diffusion coefficient is expected by cholesterol depletion. A nano-domain effect cannot, however, be dismissed completely, as it has been shown that some known lipid nano-domain proteins can show similar decreasing diffusion coefficients when depleted with MβCD [21,22]. An example is an observed decrease in diffusion of the common nano-domain marker glycosylphosphatidylinositol (GPI) by MβCD treatment [21]. More dominant factors can be to blame for the reduced diffusion coefficient, i.e., changes in membrane viscosity, tension, or the actin cytoskeleton upon treatment with MβCD [23,24]. However, from this experiment, there is no evidence for any correlation between lipid nano-domains and AQP9. Based on this information, a different strategy (for example metabolic cholesterol depletion by compactin [22]) for investigating the correlation between AQP9 and lipid nano-domains should be used in future studies.

We then treated cells with CytD and LatA to study degradation of actin fence nano-domains. Both compounds inhibit actin polymerization and reduce the number of actin fence nano-domains. In cells treated with either CytD or LatA, the diffusion coefficient was found to be 0.11 ± 0.036 µm^2^/s and 0.073 ± 0.023 µm^2^/s, respectively. The effect of the drugs was inspected visually to observe if the cell appeared healthy. Images were acquired and the diffusion coefficients were calculated. The diffusion coefficient of AQP9 in cells treated with each of those two drugs did not show a significant change compared to the nontreated cells (Figure 2B). This leads us to conclude that actin fence nano-domains have no correlation to the diffusion of the AQP9 protein.

Finally, the cells were treated with hEGF. A known effect of hEGF is an increased formation of clathrin coated pits, which subsequently increases endocytosis of the hEGF receptor [25]. Addition of hEGF resulted in a significant increase in the diffusion coefficient of AQP9 to 0.15 ± 0.044 µm^2^/s. We hypothesize that the increase in the diffusion coefficient of AQP9 is an effect of the clathrin coated pits on AQP9 diffusion. The diffusion coefficient of GFP-AQP9 under the investigated conditions can be found in Figure 2B. The results from this study opens up for further studies to evaluate the effect from hEGF in more detail. 

This study shows that kICS is an excellent tool to determine the diffusion coefficient of plasma membrane proteins. Unlike other groups of AQPs, there was no evidence of nano-domain correlation with AQP9. The decreasing diffusion coefficient by MβCD cannot, however, definitively reject a possible effect of nano-domains, and asks for further studies with alternative approaches. The increased diffusion coefficient by exposure to hEGT should be studied further to reach a better understanding of the underlying cellular processes, these studies includes loss-of-function experiments and identification of diffusion type. Further investigations to be addressed in future studies include analysis in different cell types to conclude whether the observed results are general characteristics or solely for HEK-293 cells. Additionally the labelling technique should be improved by using CRISPR-Cas9 knock-in strategies to avoid overexpression of AQP9.

## 4. Conclusions

From the kICS analysis, it can be concluded that the addition of actin polymerization inhibiting compounds CytD and LatA does not affect the diffusion coefficient of AQP9 in HEK-293 cells significantly, indicating no correlation between the actin cytoskeleton network and AQP9. Decreases in diffusion coefficients were observed when adding MβCD. These observations combined suggest that, unlike other AQPs (e.g., AQP4 [11]), AQP9 is not a nano-domain membrane protein. The effect of lipid nano-domains should, however, be accepted with care and calls for more studies. Finally, the diffusion coefficient GFP-AQP9 increased when hEGF was added, due to the EGF receptors effect on the plasma membrane. This further highlights the versatile nature of protein dynamics in the plasma membrane, and how insight into the nature of regulation of the highly heterogenous membrane is certainly required. kICS has here been shown to be a valuable tool to acquire an overview of the effects from multiple nano-domain perturbing drugs on AQP9 diffusion.

## Data Availability

Not applicable.
